# Stay in Risk Area: Place Attachment, Efficacy Beliefs and Risk Coping

**DOI:** 10.3390/ijerph19042375

**Published:** 2022-02-18

**Authors:** Chen Qing, Shili Guo, Xin Deng, Wei Wang, Jiahao Song, Dingde Xu

**Affiliations:** 1 College of Management, Sichuan Agricultural University, Chengdu 611130, China; qingchen@stu.sicau.edu.cn (C.Q.); wangwei@sicau.edu.cn (W.W.); songjiahao@sicau.edu.cn (J.S.); 2 China Western Economic Research Center, Southwestern University of Finance and Economics, Chengdu 610074, China; guoshili@swufe.edu.cn; 3 College of Economics, Sichuan Agricultural University, Chengdu 611130, China; dengxin@sicau.edu.cn; 4 Sichuan Center for Rural Development Research, College of Management, Sichuan Agricultural University, Chengdu 611130, China

**Keywords:** place attachment, self-efficacy, response efficacy, evacuation intention, relocation intention, earthquake

## Abstract

For residents living in earthquake-threatened areas, choosing suitable risk coping behaviors can effectively reduce the loss of family life and property. However, some residents still choose to continue to live within areas at risk of earthquake disaster. Place attachment may play an important role. Based on place attachment theory and the Protective Action Decision Model (PADM), this study explores the possible relationships among place attachment, efficacy beliefs, and evacuation/relocation intention. Furthermore, it examines the mediating role of efficacy beliefs. The study also used the partial least squares method (PLS-SEM) to test samples of 327 rural households in Wenchuan and Lushan earthquake-hit areas. The results show that: (1) Place attachment has a significant effect on response efficacy (RE), but not on self-efficacy (SE); (2) Place dependence (PD) has a negative and significant effect on relocation intention (RI) but has no significant effect on evacuation intention (EI). However, place identity (PI) can indirectly affect EI by influencing RE; (3) Efficacy beliefs have a significant positive effect on EI and RI. These results help us understand the complex relationships among place attachment, efficacy beliefs, and residents’ risk coping behavior, thus providing decision-making references for the formulation and improvement of regional disaster risk management policies.

## 1. Introduction

In the past two decades, a total of 7348 natural disasters occurred around the world, resulting in 1.23 million deaths, affecting the jobs, property, and health of about 4 billion people, and causing a loss of $2.97 trillion to the world economy [1]. In the face of more frequent and intense natural disasters, all we can do is prevent and respond correctly. In fact, after the disaster, many residential areas still face the threat of disaster. For example, after an earthquake disaster occurs, it will be accompanied by a series of secondary disasters such as collapses, landslides, and mudslides [2,3]. However, many residents living in earthquake-threatened areas, even in the face of disasters or secondary disasters, are unwilling to evacuate or relocate [4,5,6]. Why does this phenomenon occur? In this case, it becomes essential to understand how people make decisions when dealing with risks.

Risk-coping behaviors refer to people’s behaviors in response to natural disaster risks. Place attachment is undoubtedly a critical explanatory factor when understanding people’s response to natural disasters [7,8]. Place attachment refers to a positive emotional bond between people and a particular place [9], one of the most important psychological factors in the relationship between people and location [10]. Although economic and social factors tend to dominate behavior choices, more and more literature shows that place attachment has many psychological benefits [11,12], affecting residents’ coping behaviors. Evacuation and relocation are the two most common coping behaviors for people to respond to earthquake shocks. Both of them have been proven to be effective in reducing disaster losses [13,14]. Evacuation refers to the emergency transfer of people from a dangerous area to avoid or reduce the impact of a disaster; under normal circumstances, they can return to the original area after a certain period [15]. Relocation refers to people moving out of dangerous areas to avoid or reduce the long-term impact of disasters. It is difficult to predict the time of their return to the original area, or if they will ever return to their original place of residence [16]. Although there is a difference in essence between evacuation and relocation, they are both actions away from the risk area, except that one is temporary and the other is permanent. According to the current empirical analysis results, scholars generally believe that residents’ attachment to place will hinder them from staying away from risk areas. For example, Lavigne et al. [17] studied Indonesians living near volcanoes and found that their attachment to the place explained the failure of the evacuation plan in the form of cultural beliefs. Boon [18] investigated rural residents’ perceptions in Australia before and after flood disasters. They found that residents are reluctant to relocate even if they have experienced multiple floods; the stronger the flood victims’ attachment to place, the more difficult it is for them to accept relocation. Similarly, in a study on the behavior of Norwegian residents facing the risk of oil spills, a strong sense of place was found to be negatively related to the intention to relocate [19]. In these studies, place attachment as a barrier affects residents’ response to risk.

In addition to directly affecting residents’ risk response decisions, place attachment is also considered to be related to residents’ perceived risk. On the one hand, some studies have shown that there is a positive connection between place attachment and risk perception. For example, Stain et al. [20] studied people who have suffered from drought for a long time and found that people’s strong sense of place increases their worries about drought. On the other hand, some studies point out that there is a negative connection between place attachment and risk perception [21,22]. However, Bernardo [23] classified Portugal’s different types of risks into different levels, and found that place attachment helps to amplify the perception of high-probability risk (low risk), while weakening the perception of low-probability risk (high risk). Although these findings are contradictory, they reflect that there is indeed a close connection between place attachment and perceived risk. Perceived risk describes how a person assesses his possibility of facing threats [24], and the perceived efficacy reflects his assessment of his ability to avoid threats [25]. They are the main concern in the discussion of factors affecting natural disaster response [26,27]. Since place attachment is significantly related to perceived risk, is there a connection between place attachment and perceived efficacy? As Relph [28] asserted, if a person feels that they belong to a place, they will feel safe rather than threatened, closed rather than exposed, and relaxed rather than tense. It stands to reason that the sense of security can derive from the reduction of risk perception or the improvement of efficacy beliefs. However, so far, few studies have systematically analyzed the relationship between place attachment and efficacy beliefs. Twigger-Ross et al. [29] pointed out that place identity arises when people think that the environment is easier to manage and therefore easier to integrate into their self-conceptualisation. In other words, if the place is integrated into the identity, it means that the place can bring people a sense of particularity, continuity, self-esteem, and self-efficacy [30]. At the same time, considering the close connection between place attachment and risk coping behavior, we immediately put forward a conjecture: if there is a connection between place attachment and efficacy beliefs, can place attachment affect residents’ risk-coping behavior through efficacy beliefs? Furthermore, is the mechanism of action for the two different risk-coping behaviors of evacuation and relocation the same?

In the existing studies, scholars have carried out a great amount of research on the influence of place attachment on residents’ risk coping behavior. The current study results reveal the crucial role of place attachment in the decision of residents to stay in risk areas, but there are still limitations. It is mainly reflected in the following three aspects: Firstly, from the perspective of the research area, the previous research primarily concentrated on typically developed countries, such as the United States [31], Norway [19], Australia [18], etc., while less attention was paid to developing countries. At the same time, compared with flooded areas [32,33], volcano threatened areas [17,34], and hurricane threatened areas [35], existing studies have paid less attention to earthquake threatened areas. As a natural disaster, an earthquake can not only cause various damages itself, but also form a disaster chain and induce various secondary disasters [36]. The occurrence of these disasters will continue to reconstruct the place attachment of the residents in the disaster-threatening area [37]. Therefore, more research needs to be conducted on earthquakes. Secondly, from the perspective of research content, although many works of literature have explored the relationship between place attachment and residents’ risk coping behavior, existing research has mainly focused on a particular behavior of residents, such as disaster preparedness [38], evacuation [7], and relocation [39], and lacked simultaneous attention and comparison of two or more risk-coping behaviors. For example, evacuation and relocation are two widespread coping behaviors when people respond to earthquake disasters. However, there is a difference between these two behaviors, with evacuation resulting in a return to the area of origin, whereas relocation usually does not. So, in the same area, would residents with a stronger attachment to the place tend to choose to evacuate rather than relocate? Therefore, it is vital to simultaneously focus on the relationship between place attachment and these two coping behaviors. In addition, existing research primarily discusses the role of perceived risk in place attachment and risk-coping behavior [40,41], while there is almost no attention to the efficacy beliefs, another important psychological factor in the decision-making process of residents’ risk coping behavior. Thirdly, from the perspective of research methods, most studies usually use conventional regression analysis (such as Logit/OLS) [39]. Still, considering the complexity of the interaction between variables, conventional regression results may ignore the hidden relationship between variables. The structural equation model (SEM) provides a conceptual modeling and verification process for the many difficult-to-measure concepts involved in the study and can evaluate multi-dimensional and interrelated relationships [42,43]. Therefore, the use of structural equation models can help us better analyze the complex relationship between place attachment and coping behavior.

In this context, this study focuses on the residents in the earthquake-prone areas of Sichuan Province, introduces efficacy beliefs into the study of place attachment and residents’ risk coping behaviors, tries to build a new analysis framework, and verifies it by partial least squares method (PLS-SEM). Our research attempts to understand the complex relationship among place attachment, efficacy beliefs, and two different risk coping behaviors (evacuation and relocation). To this end, this research aims to solve the following problems:

(1) What are the characteristics of place attachment, efficacy beliefs, and risk coping behavior of residents in the earthquake-prone areas?
(2) What is the mechanism of place attachment and efficacy beliefs in residents’ disaster risk coping behavior in earthquake-prone areas?

The sections in this study are arranged as follows: Section 2 introduces the literature review; Section 3 introduces the research model and hypothesis; Section 4 introduces the method of testing the research model; Section 5 introduces the research results; Section 6 introduces the key findings, research contributions, and research limitations.

## 2. Research Background

### 2.1. Theory of Place Attachment

Additionally, related to place attachment are concepts such as place dependence, place identity, and sense of place [44], which are collectively referred to as the theory of place attachment here. Many scholars have summarized and identified the connotation of these concepts and the relationship between them [45,46,47,48]. Although there are still many disagreements, place attachment is usually the most explored [34,49,50]. Quantitative methods to measure place attachment are favored by researchers in environmental psychology. Scholars often divide place attachment into different dimensions, and then design a Likert scale to measure each dimension. Among them, the classic study of Williams et al. [51] believes that place attachment is a two-dimensional construct, which has its own potential sub-constructs, such as place identity (PI) and place dependence (PD). PI refers to the symbolic meaning given to a place when a person invests in the place psychologically. PD refers to the functional dependence caused by the particularity of the site, the uniqueness of the facility, and other characteristics. Usually, the functional bond is referred to as PD, while the emotional bond is termed PI [52].

With the development trend of research crossover, place attachment is more often used as an application tool to reveal the occurrence mechanism of some phenomena. Li et al. [53] studied the phenomenon of return after the evacuations of Hurricane Katrina and found that the subjects all expressed their strong attachment to New Orleans; this attachment to the place prompted them to decide to return. In the field of disasters, scholars have noticed that residents living in disaster-risk areas are unwilling to evacuate or relocate quickly because of their deep emotional connection with the disaster area [54]. However, what mechanism does place attachment use to influence individual coping behaviors? Is it a direct or indirect impact? What is the effect on different coping behaviors? These issues are rarely involved.

### 2.2. From Protection Motivation Theory (PMT) to Protective Action Decision Model (PADM)

PMT was proposed by Rogers et al. [55] and is a theory that is used for studying human health behavior. There are two core processes in PMT’s, i.e., threat appraisal and coping appraisal. Threat appraisal refers to the possibility that a person perceives a risk, and coping appraisal refers to the ability to perceive themself to avoid being harmed by a threat. Because of the introduction of the concept of coping appraisal, PMT exhibits unique value [27]. Response efficacy (RE) and self-efficacy (SE), as essential components of coping appraisal, have also received more and more attention [25,56,57]. Tang et al. [58] investigated the factors affecting residents’ intention of disaster prevention behavior after the Taiwan earthquake and found that RE and SE were significantly related to behavioral intention.

In the field of disaster risk management, Lindell, a well-known scholar, proposed PADM [59], which provides a complete framework for understanding people’s risk-coping behavior. Later, Lindell et al. [60] further modified the PADM. The revised model identifies three core perceptions, namely threat perceptions, protective action perceptions, and stakeholder perceptions. They form the basis for decisions about responding to an imminent or long-term threat. Among the model, efficacy belief is also an important attribute in PADM [61]. The PADM’s concept of hazard-related attributes (known as outcome efficacy) is similar to, but more extensive than, the PMT’s RE. In addition, while the PADM’s concept of resource-related attributes is markedly different from PMT’s SE, an emphasis on SE can be useful when focusing on a single protection action. In recent years, scholars have found that PADM is strongly applicable for studying people’s disaster-coping behavior, especially for earthquake disasters [62,63,64]. Van Valkengoed et al. [65] conducted a series of meta-analyses of data from 106 studies from 23 different countries. They found that the key components of PADM (outcome efficacy and self-efficacy) were both important predictors of adaptive behavioral intent. However, it is not yet clear how RE and SE affect coping behavior individually, and there is a lack of comparison between the two predictive abilities.

## 3. Research Model and Hypotheses

Consistent with Williams et al. [51], our study suggests that people’s attachment to place is based on the following two factors, i.e., PD and PI. In fact, a person may be attached to a place but does not identify with it (for example, a person likes to live in a place and wants to stay there but feels that the place is not part of his identity). Due to PI having a sensory dimension, it takes a long time to experience and feel to produce the sense and symbolic meaning related to the place [30]. In other words, the degree and duration of PD will further affect PI. Therefore, the research hypothesis is as follows:

**Hypothesis** **1** **(H1).** *PD has a positive significanteffect on PI*.

Research on place attachment and efficacy beliefs is limited in the context of disaster risk management. According to Twigger-Ross et al. [29] on place and identity processes, place attachment can produce a greater sense of SE because the environment preserves self-perception. Wang et al. [66] investigated the place attachment and disaster preparedness of residents in Shandong, China. They found that place attachment is highly correlated with SE, and SE plays a mediating role between place attachment and disaster preparedness. Thus, familiarity and attachment to a place may make people feel unique [67], resulting in positive evaluations of place control and self-competence [68]. However, few studies have linked place attachment to RE. We believe that the sense of control brought about by high levels of place attachment [69] makes residents believe that coping measures are effective. Therefore, the following research hypothesis is proposed:

**Hypothesis** **2** **(H2).** *PD has a positive significant effect on RE*.

**Hypothesis** **3** **(H3).** *PI has a positive significanteffect on RE*.

**Hypothesis** **4** **(H4).** *PD has a positive significant effect on SE*.

**Hypothesis** **5** **(H5).** *PI has a positive significanteffect on SE*.

SE refers to a person’s confidence level in their ability to take an actual protective response. In contrast, RE refers to the belief that protective actions are actually effective and can protect themselves or others from risk [27]. Existing studies often use them as predictors of risk-coping behavior [58,70] but rarely explore the relationship between them. It’s easy to imagine that people feel more confident that they can successfully deal with risks by perceiving options for effective coping. Therefore, the following research hypothesis is proposed:

**Hypothesis** **6** **(H6).** *RE has a positive significanteffect on SE*.

Efficacy beliefs include perceived SE and RE [25], consistent with perceived danger, and both are essential components of psychological factors in risk coping [55]. The higher the level of efficacy beliefs, the stronger the individual’s confidence in their own coping ability. Newnham et al. [71] evaluated Hong Kong residents’ SE and evacuation barriers. They found that residents who reported high levels of SE had fewer perceived barriers to evacuation and had more potential to prepare for evacuation. Samaddar et al. [72] investigated the evacuation willingness of residents in flooded areas of Mumbai and found that individuals with a high SE were more inclined to evacuate. Consistently, Bradley et al. [73] proposed a model of pro-environmental behavior induced by climate change and found that RE can predict environmental protection behavior in the face of environmental threats. Therefore, the following research hypothesis is presented:

**Hypothesis** **7** **(H7).** *RE has a positive significant effect on EI*.

**Hypothesis** **8** **(H8).** *RE has a positive significant effect on RI*.

**Hypothesis** **9** **(H9).** *SE has a positive significant effect on EI*.

**Hypothesis** **10** **(H10).** *SE has a positive significant effect on RI*.

Place attachment is considered a positive emotional bond between people and a specific place, characterized by the desire to maintain intimacy with the attachment object [46]. As Fried [74] noted, people who are forced to relocate are experiencing a period of mourning, just as people experience when important people die. Unfortunately, when a place is threatened by a disaster, it is often necessary to evacuate/relocate to deal with this risk [75,76]. However, people may choose to stay in the threatened area in order to avoid an interruption in their relationship with the place. This is also supported by a lot of research [8,22]. In addition, strong place attachment is believed to promote the behavioral adaptation of residents [57]. At the same time, high attachment to place is often accompanied by optimism bias (shown in people’s belief that bad events are unlikely to happen there or their high confidence that they can overcome difficulties), because it protects their identity and reduces negative emotions such as fear and anxiety [77,78]. Therefore, the following research hypothesis is presented:

**Hypothesis** **11** **(H11).** *PD has a negative significant effect on EI*.

**Hypothesis** **12** **(H12).** *PI has a negative significant effect on EI*.

**Hypothesis** **13** **(H13).** *PD has a negative significant effect on RI*.

**Hypothesis** **14** **(H14).** *PI has a negative significant effect on RI.*

We believe that efficacy beliefs play a mediating role between place attachment and coping behavior based on the above analysis. Therefore, the research hypothesis as follows:

**Hypothesis** **15** **(H15).** *PD indirectly affects EI by influencing RE*.

**Hypothesis** **16** **(H16).** *PD indirectly affects EI by influencing SE*.

**Hypothesis** **17** **(H17).** *PD indirectly affects RI by influencing RE*.

**Hypothesis** **18** **(H18).** *PD indirectly affects RI by influencing SE*.

**Hypothesis** **19** **(H19).** *PI indirectly affects EI by influencing RE*.

**Hypothesis** **20** **(H20).** *PI indirectly affects EI by influencing SE*.

**Hypothesis** **21** **(H21).** *PI indirectly affects RI by influencing RE*.

**Hypothesis** **22** **(H22).** *PI indirectly affects RI by influencing SE*.

**Figure 1:**
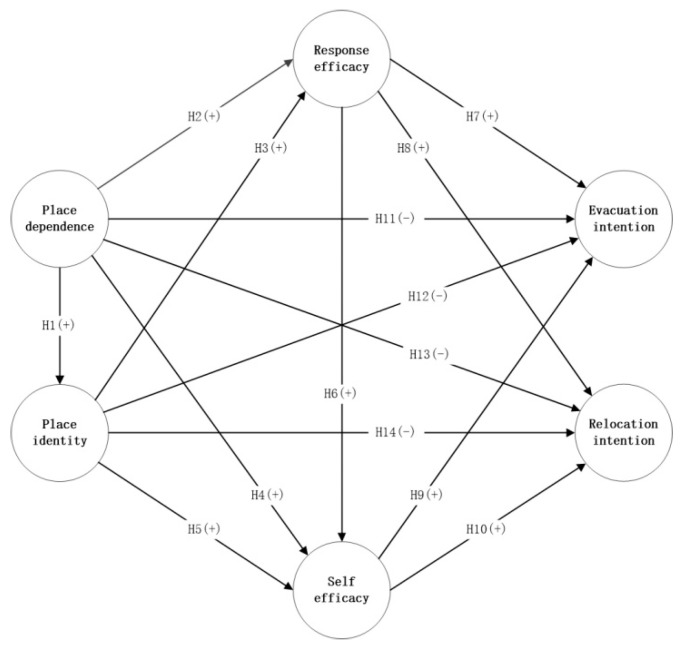
Conceptual framework.

Therefore, Figure 1 is the conceptual framework of this paper.

## 4. Method

### 4.1. Study Site

Sichuan Province is located in southwest China. As it is located in the north–south seismic belt, it is prone to earthquakes. According to CNSB (2019) [79], from 2008 to 2018, there were 19 earthquakes of magnitude five or above in Sichuan, with a total casualty of about 460,000 people and a direct economic loss of 856.8 billion Yuan caused by these earthquakes. Among them, the 12 May Wenchuan earthquake in 2008 (8 on Richter Scale) and the 20 April Lushan earthquake in 2013 (7 on Richter Scale) caused severe casualties and considerable losses to the houses and property of local residents [80]. Since it is of little practical significance to pay attention to farmers’ risk-coping behaviors and their driving mechanisms in areas where earthquake disasters are unlikely to occur, the disaster areas affected by the Wenchuan earthquake and Lushan earthquake were selected as the representative study areas in Sichuan Province. Considering the significant differences in economic development between counties involved in the two earthquakes, Beichuan and Pengzhou were selected from 10 counties in the hardest-hit areas of the Wenchuan earthquake, and Lushan and Baoxing were selected from 6 counties in the hardest-hit areas of the Lushan earthquake. It is worth noting that although many years have passed since the two major earthquakes occurred, aftershocks and a series of secondary disasters continue to occur in the region [81,82]. Therefore, we believe that the place attachment of the residents living in this area is constantly being reconstructed. Considering that the changed place attachment of residents may have a new impact on their evacuation intention (EI) and relocation intention (RI), the choice of the study area in this research is meaningful.

### 4.2. Sample

The data used in this study are from a questionnaire survey conducted by our research group comprising farmers in the hard-hit areas of the Wenchuan and Lushan earthquake in July 2019 [83]. The main contents of the survey are (1) basic information of farmers, including gender, age, disaster experience, etc.; (2) farmers’ perception of disaster risk; (3) farmers’ place attachment; (4) farmers’ disaster preparedness behavior. The survey samples were obtained by stratified random sampling [84,85]. To be specific, firstly, according to the level of economic development, two sample counties were selected from the hard-hit areas of the Wenchuan earthquake and Lushan earthquake. Secondly, according to the degree of disaster and the level of economic development of the towns, two sample towns were selected from each sample county. Thirdly, according to the distance from the village to the town government and the level of economic development, two sample villages were selected from each sample village. Finally, according to the preset random number table, 20–23 farmers were randomly selected from each sample village as sample farmers. Under the leadership of the village cadres, the trained investigators conducted one-on-one interviews at farmers’ homes. After data screening and excluding questionnaires with missing data and inconsistencies, a total of 327 valid farmer questionnaires were obtained in four districts, counties, eight townships and 16 villages.

### 4.3. Measures

The measurement of place attachment draws on Williams et al. [51], Prayag et al. [86], Raymond et al. [87], Vaske et al. [88]. These studies believe that PD and PI are parallel concepts, and they are both subordinate to the concept of place attachment. At the same time, considering that the object of this study is the farmers in the poor areas in western China, their education level is generally low. Therefore, the study modified part of the items in the classic research model to ensure that the farmers could correctly understand the meaning of the items.

As mentioned earlier, efficacy beliefs refer to an individual’s assessment of their own ability to respond to threats. Concerning existing research on the measurement methods of efficacy beliefs [25,27,89], combined with the characteristics of the data obtained from the questionnaire, this research mainly designs items to measure the efficacy beliefs from two aspects of SE and RE. It is worth noting that residents have various coping behaviors in the face of disaster threats. Correspondingly, residents have different perceptions of the effectiveness of these coping behaviors [90,91]. Therefore, in the measurement of RE, a clear coping behavior should be selected. Evacuation has been proven to reduce the impact of earthquake disasters effectively [92], so we measured the RE by measuring the effectiveness of the evacuation perceived by residents.

EI and RI are the core dependent variables of this research. According to the study of Xu et al. [39], residents’ willingness to evacuate or relocate are affected by their neighbors and the government. In order to make the measured EI/RI as lose to the actual EI/RI as possible, this study comprehensively measures residents’ EI and RI under a variety of circumstances, such as voluntary, government compulsory, and government subsidy.

All structures were measured using the Likert five-level scale, and the specific items can be found in Table 1. Among them, the items of place attachment and efficacy beliefs include: strongly disagree (1), disagree (2), generally (3), agree (4), strongly agree (5); The items of EI and RI include: very unwilling (1), unwilling (2), generally (3), willing (4), very willing (5).

### 4.4. Data Analysis

The study uses Partial Least Squares (PLS) to test the research model. PLS is a type of Structural Equation Modeling (SEM), which can integrate measurement models and structural models [93]. The measurement model examines the hypothetical connection between the indicator and the underlying structure. In contrast, the structural model estimates the hypothetical path between the exogenous (independent) and endogenous (dependent) underlying structures. In addition, PLS is often used in the analysis of complex models and is suitable for exploratory research. In this research [94], we use SmartPLS 3.0 to model the PLS path.

## 5. Results

### 5.1. Descriptive Analysis

#### 5.1.1. Demographic Characteristics

Table 2 shows the basic demographic characteristics of the research subjects. According to Table 2, 54% of the respondents are male, 46% are female, and 77% are over 45 years old. The education level of the respondents is generally low, less than 3% of the respondents have a college degree, and even 10% of the respondents have no educational experience.

#### 5.1.2. Characteristics of Place Attachment, Efficacy Beliefs and Risk Coping

**Figure 2:**
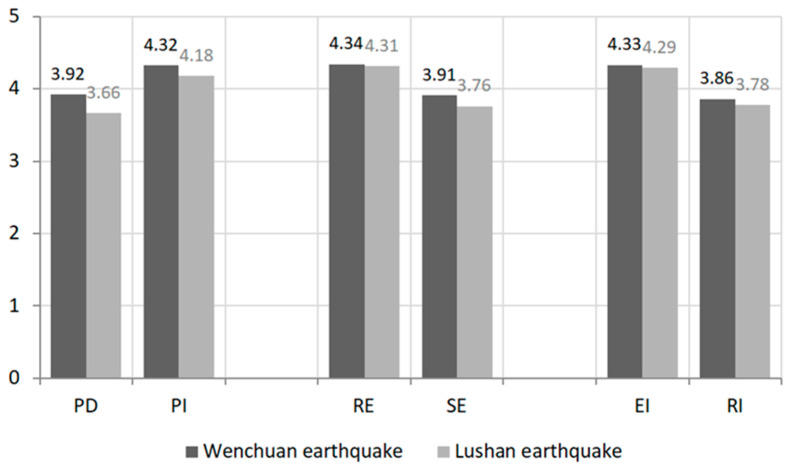
Place attachment, efficacy beliefs and risk coping characteristics of farmers in earthquake-threatened areas.

Figure 2 displays the results of a descriptive statistical analysis of all variables. Overall, farmers in the hardest-hit areas of the Wenchuan earthquake and Lushan earthquake showed similar characteristics of place attachment, efficacy beliefs, and risk coping. Based on the visual analysis of the data, the following characteristics can be found. Firstly, compared with PD, farmers have a higher level of PI. This suggests that farmers in earthquake-threatened areas feel that the place they currently live is part of their identity and have a higher sense of identity. However, the dependence on the village is not high. Secondly, the RE score of farmers is higher than the SE score, which means that farmers have a stronger perception of the effectiveness of coping behavior but a weaker perception of their own coping ability. Finally, the EI of farmers in the two earthquake-stricken areas is significantly higher than the RI. This shows that farmers are more willing to choose evacuation rather than permanent relocation when faced with the threat of earthquake disaster.

### 5.2. Research Model

#### 5.2.1. Assessment of Measurement Model

The study evaluated the effectiveness of the construct based on item loadings, Cronbach’s alphas (α), composite reliability (CR), and Average Variance Extracted (AVE) (Table 3). Firstly, the factor loadings range of the measured variables is 0.637–0.925, higher than the 0.5 recommended by Hair [95]. Secondly, for each construct, we use α and CR to measure the internal reliability of the latent variable. α is higher than 0.6, CR is higher than 0.8, indicating that the constructs have good internal consistency. Thirdly, the convergence validity of the measurement model is estimated according to AVE, and the AVE of each latent variable is greater than 0.5, indicating that the constructs have good convergence validity. In addition, discriminant validity shows the degree of correlation between each construct. Fornell et al. [42] suggested that the average variance must be higher than the variance shared between one construct and other constructs to show good discriminant validity. As shown in Table 4, the diagonal value (in bold) is greater than the value in its column, indicating that each of model construct is unique and distinct from other constructs.

#### 5.2.2. Assessment of Structural Model

Table 5 shows the path coefficients between the latent variables, the research hypothesis, and the bootstrap T statistic. According to Hair et al. [96], in the 90% confidence interval, the acceptable T statistic must be greater than 1.65. It can be seen from Table 5 that when the T statistic is less than 1.65, the hypothesis is rejected.

**Figure 3:**
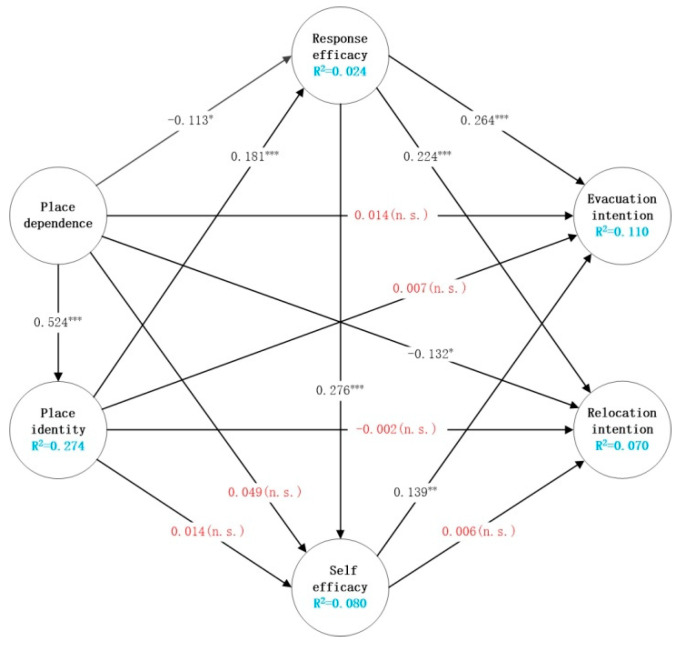
Test of research model. Note: *** *p* < 0.01; ** *p* < 0.05; * *p* < 0.1; n.s. not significant.

The path coefficient’s direction, strength, and significance level are the key factors for testing the research hypothesis. As can be seen from Figure 3, PD has a positive and significant impact on PI, which shows that the deeper the degree of PD, the more farmers will be able to identify with the place. Therefore, H1 is supported. Both PD and PI have significant effects on RE, but the effect of PD on RE is negative, which is inconsistent with our initial hypothesis. That is, a higher sense of PD leads to a lower perception of RE. This may be because the subjects in the study are older, with an average age of 53 years old. These people tend to be local natives who have lived in the area over a long period and have a strong sense of dependence on the village [67]. However, due to their low years of education and limited exposure to disaster response information, their perception of the effectiveness of response measures was inadequate. So H2 is rejected, and H3 is supported. However, the effect of place attachment on SE is not significant, H4 and H5 are rejected. In addition, RE has a significant positive impact on SE. Through the perception of the effectiveness of coping measures, farmers can improve their confidence in coping with risks. Therefore, H6 is supported. Moreover, RE has a significant positive impact on EI and RI. That is, farmers who have a higher perception of the effectiveness of the measures will be more willing to evacuate or relocate. H7 and H8 are supported. SE only has a positive and significant effect on EI but has no significant effect on RI. Therefore, H9 is supported, and H10 is rejected. Finally, place attachment has no direct effect on EI, H11 and H12 are rejected. However, PD has a direct negative impact on the willingness to relocate, but the significance of the path coefficient is low. This shows that farmers’ high dependence on local areas will reduce their willingness to relocate. Furthermore, H13 is supported, and H14 is rejected.

#### 5.2.3. Mediation Analyses

In the literature [97,98], efficacy belief is often added to models as a mediating variable. Therefore, we believe that place attachment can influence residents’ risk coping behavior through efficacy beliefs. So, this section shows the mediating role of efficacy beliefs. Based on the results of the bootstrap method, Table 6 reports the indirect effects of RE and SE as mediators of place attachment and two coping behaviors. From Table 6, we can see that only two mediation paths are significant. That is, PI can indirectly affect EI and RI through its impact on RE, H19 and H21 are supported. This indicates that although PI does not directly affect EI and RI, farmers’ high level of PI can increase their willingness to evacuate and relocate by promoting the effectiveness of their perceived response measures. In addition, because it is unclear whether the direct impact of PI on EI and RI is positive or negative, it is impossible to judge whether the response effectiveness plays a role in magnifying or minimizing the direct impact. Moreover, since it is not clear whether the direct impact of PI on EI and RI is positive or negative, it is impossible to judge whether RE plays a role in magnifying or reducing the direct influence.

## 6. Discussion and Conclusions

The connection between place attachment and risk coping strategy, especially residents’ adaptive behavior when facing disaster threats, is a hot topic in many current studies [8,99]. Compared with the existing studies, this study mainly makes the following three contributions: Firstly, combining place attachment theory and PADM, this research builds a theoretical analysis framework for residents’ place attachment, efficacy beliefs, and risk coping behavior, and builds a corresponding indicator system; Secondly, the study also focused on the two risk coping behaviors of evacuation and relocation, compared the differences in the effects of place attachment and efficacy beliefs on EI and RI, and provided evidence to explain the different risk-adaptation behaviors of residents; Thirdly, the study successfully proved the mediating role of RE and explored the relationship among place attachment, efficacy beliefs, and coping behavior of residents in the earthquake-threatened area of Sichuan Province.

This study aims to explore how place attachment and efficacy beliefs can be combined to predict residents’ risk coping behaviors. At the same time, the risk coping behaviors of residents are classified as evacuation and relocation, and further explore the decisive factors that affect residents’ intention to evacuate and relocate. The research results show that, first of all, consistent with the results of Vaske et al. [88] and Su et al. [100], there is a close relationship between PD and PI. PD precedes PI, and the emergence of PD can enhance the identity of place. Secondly, place attachment significantly impacted on RE, but it had no significant impact on SE. This is inconsistent with research conducted by Wang et al. [66] on typhoon and flood-threatened areas. They found that place attachment has a positive and significant effect on SE. This may be because the disaster concerned in this study is earthquake. Compared with typhoon and flood disasters, earthquake disasters are sudden and hugely destructive [101]. Even though farmers’ familiarity and attachment to the place have produced control over the place, this sense of control still cannot support farmers to make a positive assessment of their own ability to cope with the earthquake. Then, as predicted, RE had a positive and significant effect on SE. Moreover, PD had a significant negative impact on RI but no significant impact on EI. Consistent with the thinking of Ariccio et al. [7], this may be because there is a close connection between the home and the evacuation site (usually only a few minutes to walk). People may think that there is continuity between the home and the evacuation site instead of opposition; therefore, leaving home to the place of evacuation does not pose a threat to place attachment. Nevertheless, relocation means moving away from the place of residence to another place, which obviously affects the place attachment, so it presents this result. In addition, the study also discovered the mediating role of RE. That is, PI can indirectly affect evacuation and relocation intentions by affecting the RE. We believe that RE is one of the most important cognitive variables that connect people’s understanding of risk and their intention to take action. Although the action intention does not necessarily lead to the actual response behavior [27], it is crucial that the action intention is first formed during the entire disaster response process [60]. Finally, consistent with relevant research results [58,65,102], efficacy beliefs positively and significantly affected EI and RI. This shows that RE and SE, as significant predictors, have a relatively large explanation for people’s adaptive behavior intention when responding to earthquake disasters. This further confirms the accuracy of the PADM.

In recent years, the Chinese government has gradually developed strong national-disaster preparedness and disaster relief capabilities, including a strong monitoring system [103,104,105], which can detect the occurrence of natural disasters in time and quickly dispatch emergency relief supplies to disaster-stricken areas [106,107,108,109]. However, despite the strong disaster preparedness and relief capabilities at the national level, the provincial or county level, especially in poor rural areas, is relatively weak in resisting disaster risks. In this case, this study provides some enlightenment for developing an emergency response in poor rural areas. For example, this research found that the strong PD of farmers is one of the reasons that hinders them from taking correct and effective response measures, especially in their acceptance of relocation. This finding affirms the importance of studying man-land relationships and helps policymakers better understand and intervene in people’s behavior in response to disaster threats. On the one hand, we need to continue to step up advocacy campaigns to popularize the effectiveness of disaster prevention measures, encourage residents to protect themselves in emergencies, and pass on the knowledge of disaster reduction to families and communities [110,111,112]. On the other hand, it is necessary to consider the residents’ special feelings towards the place and fully respect the relationship between people and place. For example, for residents with strong local attachment and low willingness to relocate, policies should be formulated to guide them to make perfect disaster preparedness instead of forced relocation.

This study still has some shortcomings, which can be further explored in future research. For example, residents have different perceptions of the effectiveness of evacuation and relocation. However, the measurement of RE in this article only includes three questions about evacuation, and the RE variable constructed in this way may not be objective and accurate. Additionally, this study only uses cross-sectional data to explore the relationship between residents’ place attachment and their risk coping behaviors. At the same time, their place attachment (such as PI and PD) may change dynamically [48,113], and panel data is needed to reveal better the impact of place attachment on residents’ evacuation and relocation behaviors. In addition, it may be useful to explore the possible relationship between age and place attachment as much of the sample was older. It is reasonable that elders with a longer-standing connection to the land may have unique levels of place attachment compared with others. Finally, although this study uses quantitative analysis methods to reveal the mechanism of place attachment and efficacy beliefs on residents’ risk-coping behaviors, it cannot automatically obtain a reasonable explanation for the phenomenon that people still stay in disaster risk areas in reality. For example, some social conditions such as class, SES, or wealth/resources also play an important role in coping behavior. Practical obstacles (such as lack of time, money, knowledge, or social support) due to these factors may affect efficacy, evacuation, or relocation. Therefore, qualitative methods can be introduced in the next study to make the interpretation of the results more convincing.

## Figures and Tables

**Table 1:** Variable measurement items.

Constructs	Code	Items	Mean	SD
Place dependence	PD1	I never thought about moving out of the village and living somewhere else	3.37	1.51
	PD2	I don’t want to move from here because I’m used to the way of life here	4.06	1.06
	PD3	Although I am afraid of disasters, I still don’t want to move away from here, because my ancestors have been here, and my roots are here	3.91	1.17
Place identity	PI1	Living in this village makes me more satisfied than living in other places	4.02	1.03
	PI2	It’s more comfortable in the village than in other places. I can do whatever I want, and I feel more at ease	4.41	0.81
	PI3	I prefer to stay in the village unless I go out to do errands	4.31	0.90
Self-efficacy	SE1	When the earthquake disaster strikes, I know the evacuation routes in the village	4.19	1.15
	SE2	I know the location of emergency shelters in the village	4.02	1.22
	SE3	I know the existing disaster prevention measures in the village	3.28	1.31
Response efficacy	RE1	Evacuation can effectively prevent injury and death	4.37	0.88
	RE2	If I evacuate, I can effectively avoid injury and death	4.28	0.91
	RE3	Evacuation can effectively reduce physical and psychological pain	4.33	0.91
Evacuation intention	EI1	Faced with the threat of disaster, without considering other people, if I am allowed to evacuate voluntarily, I am willing to evacuate	3.68	1.37
	EI2	Faced with the threat of disaster, if the villagers close to me have evacuated, I am willing to evacuate	4.55	0.86
	EI3	Faced with the threat of disaster, if the government forces me to evacuate, I am willing to evacuate	4.45	0.91
	EI4	Facing with the threat of disaster, if the government gives a certain subsidy, I am willing to evacuate	4.57	0.84
Relocation intention	RI1	Faced with the threat of disaster, without considering other people, if I am allowed to relocate voluntarily, I am willing to relocate	3.10	1.53
	RI2	Faced with the threat of disaster, if the villagers close to me have relocated, I am willing to relocate	4.06	1.35
	RI3	Faced with the threat of disaster, if the government forces me to relocate, I am willing to relocate	3.96	1.31
	RI4	Facing with the threat of disaster, if the government gives a certain subsidy, I am willing to relocate	4.15	1.26

**Table 2:** Demographic profile.

	Frequency	Relative Frequencies (%)
Gender		
Male	176	53.82
Female	151	46.18
Age		
14–17	3	0.92
18–25	11	3.36
26–35	17	5.20
36–45	44	13.45
46–60	149	45.57
60+	103	31.50
Education Level		
illiteracy	34	10.40
Primary	143	43.73
Middle	114	34.86
High	27	8.26
College+	9	2.75

**Table 3:** Assessment of construct validity.

Constructs	Code	Loadings	α	CR	AVE
Place dependence	PD1	0.742	0.733	0.850	0.654
	PD2	0.831			
	PD3	0.849			
Place identity	PI1	0.858	0.761	0.861	0.675
	PI2	0.745			
	PI3	0.857			
Self-efficacy	SE1	0.835	0.670	0.816	0.597
	SE2	0.763			
	SE3	0.717			
Response efficacy	RE1	0.859	0.811	0.888	0.726
	RE2	0.879			
	RE3	0.818			
Evacuation intention	EI1	0.637	0.845	0.897	0.690
	EI2	0.803			
	EI3	0.924			
	EI4	0.925			
Relocation intention	RI1	0.855	0.846	0.896	0.685
	RI2	0.725			
	RI3	0.852			
	RI4	0.869			

**Table 4:** Discriminant validity (Fornell–Lacker criterion).

	PD	PI	RE	SE	EI	RI
PD	**0.808**					
PI	0.524	**0.821**				
RE	−0.018	0.122	**0.852**			
SE	0.051	0.073	0.277	**0.772**		
EI	0.021	0.057	0.303	0.213	**0.831**	
RI	−0.137	−0.043	0.228	0.061	0.452	**0.827**

**Table 5:** Summary of hypotheses testing.

Hypotheses	Relations	Original Sample	Standard Deviation	T Statistics	*p*-Values	Sign. Level	Decision
H1	PD -> PI	0.524	0.05	10.551	0.000	***	Supported
H2	PD -> RE	−0.113	0.063	1.787	0.074	*	Rejected
H3	PI -> RE	0.181	0.068	2.636	0.008	***	Supported
H4	PD -> SE	0.049	0.073	0.671	0.502	n.s.	Rejected
H5	PI -> SE	0.014	0.063	0.224	0.822	n.s.	Rejected
H6	RE -> SE	0.276	0.056	4.954	0.000	***	Supported
H7	RE -> EI	0.264	0.069	3.823	0.000	***	Supported
H8	RE -> RI	0.224	0.069	3.257	0.001	***	Supported
H9	SE -> EI	0.139	0.062	2.248	0.025	**	Supported
H10	SE -> RI	0.006	0.059	0.094	0.925	n.s.	Rejected
H11	PD -> EI	0.014	0.064	0.225	0.822	n.s.	Rejected
H12	PI -> EI	0.007	0.071	0.105	0.917	n.s.	Rejected
H13	PD -> RI	−0.132	0.075	1.753	0.080	*	Supported
H14	PI -> RI	−0.002	0.079	0.021	0.983	n.s.	Rejected

Note: *** *p* < 0.01; ** *p* < 0.05; * *p* < 0.1; n.s. not significant.

**Table 6:** Assessment of mediation effects.

Hypotheses	Relations	Original Sample	Standard Deviation	T Statistics	*p*-Values	Sign. Level	Decision
H15	PD -> RE -> EI	−0.030	0.020	1.496	0.135	n.s.	Rejected
H16	PD -> SE -> EI	0.007	0.012	0.576	0.564	n.s.	Rejected
H17	PD -> RE -> RI	−0.025	0.017	1.506	0.132	n.s.	Rejected
H18	PD -> SE -> RI	0.000	0.005	0.052	0.958	n.s.	Rejected
H19	PI -> RE -> EI	0.048	0.022	2.141	0.032	**	Supported
H20	PI -> SE -> EI	0.002	0.010	0.200	0.841	n.s.	Rejected
H21	PI -> RE -> RI	0.040	0.020	1.984	0.047	**	Supported
H22	PI -> SE -> RI	0.000	0.004	0.020	0.984	n.s.	Rejected

Note: ** *p* < 0.05; n.s. not significant.

## Data Availability

Not applicable.

## Abbreviations

PD	place dependence
PI	place identity
RE	response efficacy
SE	self-efficacy
EI	evacuation intention
RI	relocation intention
PMT	Protection Motivation Theory
PADM	Protective Action Decision Model
